# Adolescent social networks matter for suicidal trajectories: disparities across race/ethnicity, sex, sexual identity, and socioeconomic status

**DOI:** 10.1017/S0033291721000465

**Published:** 2022-11

**Authors:** Yunyu Xiao, Michael A. Lindsey

**Affiliations:** 1 School of Social Work, Indiana University-Purdue University Indianapolis, Indianapolis, IN 46202; 2 School of Social Work, Indiana University-Bloomington, Bloomington, IN 47401; 3 McSilver Institute for Poverty Policy and Research, New York University, New York, NY, 10003; 4 Silver School of Social Work, New York University, New York, NY 10003

**Keywords:** Adolescence, family cohesion, health disparities, life course, minority, social networks, suicidal ideation, suicidal trajectories, suicide attempt, young adult

## Abstract

**Background:**

Examining social networks, characterized by interpersonal interactions across family, peer, school, and neighborhoods, offer alternative explanations to suicidal behaviors and shape effective suicide prevention. This study examines adolescent social networks predicting suicide ideation and attempt trajectories transitioning to adulthood, while revealing differences across racial/ethnic, sex, sexual identity, and socioeconomic status.

**Methods:**

Participants included 9421 high school students (*M*_age_ = 15.30 years; 54.58% females, baseline) from Waves I–IV of the National Longitudinal Study of Adolescent to Adult Health, 1994–2008. Latent class growth analyses were conducted to identify suicide ideation and attempt trajectories. Multivariate multinomial logistic regressions examined the relationships between social network characteristics during adolescence and suicidal trajectories. Interaction terms between social networks and sociodemographic characteristics were included to test moderation effects.

**Results:**

Three suicidal ideation trajectories (*low-stable*, *high-decreasing, moderate-decreasing-increasing*) and two suicide attempt trajectories (*low-stable, moderate-decreasing*) were identified. Greater family cohesion significantly reduced the probability of belonging to *high-decreasing* (Trajectory 2) and *moderate-decreasing-increasing* (Trajectory 3) suicidal ideation trajectories, and *moderate-decreasing* (Trajectory 2) suicide attempt trajectory. Race/ethnicity, sex, and sexual identity significantly moderated the associations between social networks (household size, peer network density, family cohesion, peer support, neighborhood support) and suicidal trajectories.

**Conclusions:**

Social networks during adolescence influenced the odds of belonging to distinct suicidal trajectories. Family cohesion protected youth from being in high-risk developmental courses of suicidal behaviors. Social networks, especially quality of interactions, may improve detecting adolescents and young adults at-risk for suicide behaviors. Network-based interventions are key to prevent suicidal behaviors over time and suicide intervention programming.

## Introduction

Despite consistent clinical and policy efforts to reduce suicide, suicide rates in the USA increased by 46% between 2000 and 2019 from 9.1% to 13.3% among people aged 10–34 years (Centers for Disease Control and Prevention, 2020). Suicide is the second leading cause of death among adolescents and young adults. From a life course perspective, early risk and protective factors may influence suicidal behaviors in later developmental stages (Fazel & Runeson, 2020; Franklin et al., 2017). To develop more fine-grained suicide prevention that effectively targets at-risk individuals, it is important to identify precipitating factors leading to heightened suicide risks during sensitive developmental periods (Nkansah-Amankra, 2013; Rueter, Holm, McGeorgf, & Conger, 2008; Thompson & Swartout, 2018).

Prior research has predominantly focused on clinical predictors of suicidal behaviors, including mental disorders and history of suicide attempts (Cha et al., 2018a; Fazel & Runeson, 2020; Franklin et al., 2017; Glenn, Franklin, & Nock, 2015; Nock, Ramirez, & Rankin, 2019). Yet, not all youth who died by suicide have been diagnosed with depression (Nock et al., 2019). For example, social factors, such as social networks, have been understudied in relation to suicidal behaviors than the literature on psychiatric risks (Fazel & Runeson, 2020). This is particularly concerning since a recent meta-analysis of suicide risk factors observed in studies in the past 50 years suggested existing predictors provided limited explanatory power to elucidate the etiology of suicidal behaviors over time (Franklin et al., 2017). Examining social networks, characterized by interpersonal interactions across family, peer, school, and neighborhood, offer alternative explanations beyond psychiatric risk indicators that might: (1) further elucidate precursor factors influencing suicidal behaviors, and (2) shape effective suicide prevention (Wray, Colen, & Pescosolido, 2011).

The Network Episode Model (NEM) posits that social networks shape individuals' ‘*illness career*’ – i.e. the onset, course, and decisions related to one's mental illness (Pescosolido, 1991). Grounded in the NEM (Pescosolido, 1991, 1992), this study addresses social network influences during the adolescent developmental period, in particular, how this period might shape the ‘*suicidal career*’ of adolescents through their young adulthood developmental periods – i.e. long-term, personal, and socially-embedded suicidal trajectories (Michel, Dey, Stadler, & Valach, 2004). The NEM conceptualizes social networks into structure (i.e. size, density, duration, reciprocity, types of ties, frequency of content, multiplicity) and function (i.e. received and perceived social support; Pescosolido, 1991). The NEM (Pescosolido, 1991) also conceptualizes the third area of network focus, *network content*, but given the lack of availability of network content indicators in the dataset used in this study, only structure and function will be examined. Structurally, social isolation, lack of friendship, and densely connected school networks were associated with increased adolescent suicidal behaviors (Bearman & Moody, 2004; Fulkerson et al., 2006). Conversely, larger peer networks and more frequent family meals are associated with lower suicidal ideation and attempts among adolescents (Fulkerson et al., 2006; Liu, 2005). Functionally, social support during childhood has a long-lasting impact on suicidal behaviors (Nkansah-Amankra, 2013). In particular, children with poor parent–child attachment (e.g. parents hardly help talk about difficulties with the child) before 14 years old showed a greater risk of suicidal ideation during later adolescents (Fergusson, Woodward, & Horwood, 2000). Peer support and perceived school support serve as the protective factors of suicide risks (Kerr, Preuss, & King, 2006; Miller, Esposito-Smythers, & Leichtweis, 2015). Neighborhood support was also found to protect youth from suicidal behaviors, particularly among low-income adolescents (Farrell, Moledina, & Katta, 2019). In sum, these findings suggest adolescence is critical to examine social network influences across different socio-ecological contexts (i.e. family, peer, school, and neighborhood; Pescosolido, 1991).

Extant research has identified disparities in suicidal behaviors across race/ethnicity, sex, sexual identity, and socioeconomic status (SES; Baiden *et al*. 2020; Lindsey, Sheftall, Xiao, & Joe, 2019; Xiao, Romanelli, & Lindsey, 2019). For example, Black adolescents were identified as the only group showing the increasing rates of suicide attempts between 1991 and 2017 (Lindsey et al., 2019). Female adolescents consistently reported greater nonfatal suicidal behaviors than males (Cha et al., 2018a). Sexual minority (lesbian, gay, bisexual, or questioning) youth showed persistently higher risks of suicidal ideation and suicide attempts from adolescence to adulthood than heterosexual counterparts (Giano, Currin, Deboy, & Hubach, 2020). Adolescents living in lower-SES families were found to have higher suicide risks along with their development (Farrell et al., 2019).

Although existing studies started to document disparities of suicidal behaviors, three major limitations exist. First, most literature used cross-sectional design in *describing* the disparities. More research is needed, however, to understand the unique risk and protective factors of elevated suicide risk for individuals from diverse racial/ethnic, sex, sexual identity, and SES groups over multiple developmental periods. Second, previous longitudinal studies mainly focused on the prevalence and course of suicidal behaviors (e.g. Voss et al., 2019), while few captured individual heterogeneity in the incidence and progression of suicidal ideation and attempts over time. Neither of the two studies on suicidal trajectories in the USA (Thompson & Swartout, 2018) and Canada (Geoffroy, Orri, Girard, Perret, & Turecki, 2020) examined the impact of early-adolescent social networks on trajectories. Third, there is a dearth of empirical studies comprehensively examining the effects of different types of social networks from various sources (i.e. families, peers, neighborhood) on suicidal trajectories since adolescence. Even rarer are those investigating the sociodemographic differences in the associations. One exception is a study showing perceived closeness with the father significantly associated with fewer suicidal ideation for females, but not for males (Liu, 2005). In sum, little is known about how different sociodemographic characteristics moderate the effects of social network characteristics, including network structure and functions, on suicidal trajectories over time.

The purpose of this study, therefore, is to examine both social network structure (i.e. family structure, household size, peer network size, and density) and network function (i.e. family cohesion, peer support, school connectedness, neighborhood support) on suicidal ideation and suicide attempts trajectories. We are particularly concerned with how social network experiences in adolescence shape long-term suicidal risk. We also seek to identify separate trajectories for ideation and attempts, as the risk factors operate differently for each outcome (Klonsky & May, 2015; Klonsky, May, & Saffer, 2016; Klonsky, Saffer, & Bryan, 2018; May, Pachkowski, & Klonsky, 2020). A second study purpose pertains to examining how sociodemographic factors interact with social network characteristics to shape suicidal risk trajectories. This latter study's purpose is important because to develop more effective suicide prevention, it is necessary to understand *which* social network factor predicts suicidal trajectories, *whom* the social network factors pertain to, and *when* these associations are most salient (Cha et al., 2018b; Nock et al., 2019).

## Methods

### Data and sample

This study used the restricted-use data from the National Longitudinal Study of Adolescent to Adult Health (Add Health), a longitudinal study using a multistage stratified cluster design to recruit a nationally representative sample of 20 745 adolescents in grades 7th–12th from 132 schools across the USA in 1994–1995 and were followed through four surveys. Add Health used a school-based sampling design. Based on the primary sampling frame derived from the Quality Education Database, the study selected 80 high schools across urban, suburban, and rural areas with probability proportional to size, as well as 52 middle schools that were paired with the high schools as feeder schools. Feeders were replaced within each stratum until all school pairs were found. Schools were stratified by region, urbanicity, school type (public, private, parochial), ethnic mix, and size. Details of the Add Health design can be found in previous reports (Harris et al., 2019). The analytic sample of this study (*n* = 9421) included respondents who participated in the in-home interviews across Waves I (1994–1995, response rate: 79.0%, *n* = 20 745, ages 11–19 years), Wave II (1996, response rate: 88.6%, *n* = 14 738, ages 12–20 years), Wave III (2001–2002, response rate: 77.4%, *n* = 15 197, ages 18–26 years), and Wave IV (2007–2008, response rate: 80.3%, *n* = 15 701, ages 24–32 years), had the longitudinal sampling weight of Wave IV to adjust sampling design, and few missing values in the outcome variables. Response rates were calculated based on individuals in sibling pairs who were interviewed at Wave I and who were eligible for the Wave II, III, and IV samples (Harris, 2010; 2013; Harris, Halpern, & Whitsel, 2009). The increase in response rates from one wave to the next may be related to increases in the proportional number of people who were eligible. For example, 15 197 participants were interviewed in Wave III out of 19 962 eligible samples (Chantala, Kalsbeek, & Andraca, 2004), whereas 15 701 participants were interviewed in Wave IV drawn from 19 560 eligible sample of the original 20 745 Wave I respondents (ineligible respondents included 184 who moved out of the country, 87 military stationed out of the country, and 126 deceased at Wave IV). The longitudinal sampling weight was required for the analytic sample to adjust the non-response and missing data to estimate population-average models (Chen & Harris, 2020). Previous studies support that the non-responses are negligible after incorporating post-stratification sampling weights (Brownstein et al., 2010; Chantala et al., 2004).

### Procedure

Local IRB approval (IRB-FY2018-1561) was obtained to analyze the restricted-use Add Health data. The Add Health data do not contain respondent identifiers. Written informed consent was obtained from the respondents' parents for those aged 18 years and younger and respondents older than 18 years. Surveys were privately administered using Computer-Assisted Personal Interview (CAPI) and Audio Computer-Assisted Self-Interview (ACASI).

### Measurements

#### Longitudinal outcome: suicidal behaviors

Measures of suicidal ideation and suicide attempts across all four waves were used to identify suicidal ideation trajectories and suicide attempt trajectories, respectively. *Suicidal ideation* was assessed by a single-item question asking participants, ‘During the past 12 months, did you ever seriously think about committing suicide?’ (0 = *no*; 1 = *yes*). *Suicide attempt* was measured by asking, ‘During the past 12 months, how many times did you actually attempt suicide?’ (0 = *none*; 1 = *1 or more times*). Adolescents were not asked about suicide attempts in the absence of suicidal ideation.

#### Predictors: social networks during adolescence

This study includes social network variables across multiple sources (family, peer, school, and neighborhood) from Wave I when participants were adolescents. Social network structures included family-level and peer-level structures. Social network functions included family cohesion, peer support, school connectedness, and neighborhood support.

*Family-level structure* included family structure (0 = *all other arrangements*; 1 = *married, two parents*) and household size (0 = ⩽*3 people*; 1 = ⩾*4 people*). *Peer network structure* was measured by peer network size and peer network density based on the friendship nomination section. Add Health study collected network data from all students who attended each participating school by asking the respondent to nominate up to five male and five female friends from the roster of all students enrolled in the respondent's school and in the sister school. Nominated friends were further identified using a unique identification number (Carolina Population Center, 2001). Peer network size was computed as the number of friend nominations inside and outside the participant's school (range from 0 to 30). Peer network density was measured using the constructed send- and receive-network density measure of the ego (i.e. adolescent participants connected to everyone in the network) available in the Add Health data (Carolina Population Center, 2001). Eco-centered networks (e.g. social networks from ego's point of view) were composed of direct ties sent to other nominated friends and received by the participant. Density is calculated as the ratio of actual friendship ties among participants in the nominated eco-centered networks to all possible ties as indicated in the following formula (Bearman, Moody, & Stovel, 2004; Wasserman & Faust, 1994)). If few links exist among the eco-centered networks, the density values are closer to 0, whereas a completed linked network has a density score of 1.
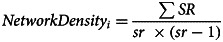
where *SR* = total ego sendandreceive network; *sr* = number of nodes in SR

*Family cohesion* was assessed by the mean values of three items: (1) family understands you, (2) family has fun together, and (3) family pays attention to you (*Cronbach's α* = 0.78). Responses were on a five-point scale (1 = *not at all*, 2 = *very little*, 3 = *somewhat*, 4 = *quite a bit*, and 5 = *very much*). *Peer support* was measured by the mean values of three items regarding the extent to which adolescents (1) feel close to people at school, (2) feel socially accepted, and (3) feel cared for by friends (Cronbach's *α* = 0.60). Answers were on a five-point scale (1 = *strongly agree*, 2 = *agree*, 3 = *neither agree nor disagree*, 4 = *disagree*, to 5 = *very much strongly disagree*). *School connectedness* was assessed using the mean scores of six youth-report items indicating the degree to which adolescents (1) felt cared for by teachers, (2) had trouble getting along with teachers (reverse-coded), (3) felt treated fairly by teachers, (4) felt safe in school, (5) felt part of their school, and (6) felt happy at school (Cronbach's *α* = 0.74). Responses were on a five-point scale (1 = *never*, 2 = *just a few times*, 3 = *about once a week*, 4 = *almost every day*, and 5 = *every day*). *Neighborhood support* was assessed using the mean score of three items asking whether adolescents, (1) knew most of the people in their neighborhood, (2) stopped on the street to talk with someone who lives in their neighborhood in the past month, and (3) thought people in this neighborhood look out for each other (0 = *not true*, 1 = *true*).

#### Sociodemographic characteristics

*Race/ethnicity* was based on two Wave I self-report questions about respondents' Hispanic or Latino origin and race. Seven racial/ethnic categories were created (1 = *non-Hispanic White*, 2 = *non-Hispanic Black*, 3 = *Hispanic*, 4 = *non-Hispanic Asians*, 5 = *non-Hispanic American Indian or Native American*, 6 = *other races*, 7 = *multiracial*). *Sex* referred to self-report biological sex (1 = *female*, 0 = *male*). *Sexual identity.* Sexual identity was measured based on the question asking participants to choose the description that best fits how you think about yourself. Original responses (1 = 100% heterosexual, 2 = mostly heterosexual, but somewhat attracted to people of your own sex, 3 = bisexual, 4 = mostly homosexual, but somewhat attracted to people of the opposite sex, 5 = 100% homosexual, 6 = not sexually attracted to either males or females) were dichotomized in the analysis (1 = *sexual minorities*, 0 = *heterosexual*). *Age* was a continuous variable, ranging from 13 to 32 years. *SES* was measured by parent-report maternal educational level (1 = *never went to school or less than high school*, 2 = *high school graduate*, 3 = *some college*, and 4 = *college graduate or higher*) and receipt of public assistance (1 = *yes*, 0 = *no*).

#### Depressive symptoms

Past-week depressive symptoms were assessed using a shortened nine-item version of the Center for Epidemiologic Studies Depression Scale (CES-D; Radloff, 1977). Responses were rated on a four-point scale (0 = *rarely or none of the time*; 1 = *sometimes*, 2 = *a lot of the time*, 3 = *most or all of the time*). Positively worded items were reverse-coded, and mean scores were calculated (Cronbach's *α* = 0.79).

### Statistical analysis

Analyses were conducted using SAS Version 9.4 (SAS Institute Inc., Cary, NC, USA), Stata version 16 (StataCorp), and Mplus version 8 (Muthén & Muthén, 1998–2017). Given the clustered nature (adolescents within schools) and stratified sampling design of Add Health, survey packages with adjusted grand sampling weights (i.e. ‘GSWGT4’) were used to produce estimates generalizable to the US population and provide robust standard error estimation. Examining missing data patterns revealed missing at random, and attrition bias was minimum (details available upon request). Multiple imputations were performed in Mplus to create 50 imputed datasets with 20 auxiliary variables (e.g. age, sex). Outlier and non-normality were investigated and adjusted.

Descriptive statistics were examined for all study variables. Rao Scott χ^2^ tests and *t* tests were used to assess differences of baseline suicidal behaviors in sociodemographic characteristics, social networks, and depression. Latent class growth analysis (LCGA) was used to characterize the distinct and unobserved subgroups representing varying trajectories of suicidal ideation and suicide attempts across Waves I–IV. Traditional longitudinal models (e.g. multilevel modeling, random-effect ANOVA) assume all individuals are drawn from a single population characterized by a single set of common parameters (e.g. means, variances) (Nagin & Tremblay, 2001). Thus, they fail to capture individual differences in growth trajectories beyond the average. LCGA is a special type of growth mixture modeling that allows for exploring differences in growth trajectories across unobserved subpopulations to identify homogeneous, mutually exclusive groups that exist within a heterogeneous population (Nagin & Tremblay, 2001). By utilizing a person-centered approach to categorize individuals into distinct groups based on individual response patterns, LCGA is more effective than traditional longitudinal methods at highlighting changes in behaviors over time (Nagin & Tremblay, 2001).

We estimated one-, two-, three-, four-, and five-class models, using age as a marker of time. We followed the recommended steps to estimate an unconstrained model without covariates and compared the model fit with the constrained model with covariates (Jung & Wickrama, 2008). To account for the possibilities of nonlinear trajectories of suicidal behaviors, we estimated linear and quadratic LCGA models and compared the nested models (e.g. linear *v.* quadratic trajectory) were examined using (1) nested χ^2^ difference test (χ^2^*_DIFF_*), and (2) model fit indices (Jung & Wickrama, 2008; Wickrama, Lee, O'Neal, & Lorenz, 2016). We chose the constrained linear models with covariates. Full information maximum likelihood estimation was used to pursue parameter estimates and model tests. The final number of trajectories was selected based on model fit indices (Nylund, Asparouhov, & Muthén, 2007), entropy (Asparouhov & Muthen, 2014), the smallest estimated class proportions, and conceptual meaning (Xiao et al., 2019). Akaike Information Criterion (AIC), Bayesian Information Criterion (BIC), and adjusted BIC were used to compare model fit, with smaller values indicating a better-fit model (Nagin & Tremblay, 2001; Nylund et al., 2007). Entropy compared how distinctly each model classified individuals into discrete suicidal behavior trajectories, with 0.60 indicating good separation (Asparouhov & Muthen, 2014; Nagin & Tremblay, 2001). Latent classes less than 1% of the sample or did not converge were not considered due to poor generalizability (Xiao et al., 2019). The Lo-Mendell-Rub adjusted likelihood ratio test (LMR-LRT) tested whether a model with one more class significantly improved model fit relative to a model with fewer classes, indicated by a *p* value <0.05 (Asparouhov & Muthen, 2014; Nagin & Tremblay, 2001).

After determining the suicidal trajectories, we conducted bivariate analyses to identify correlates of suicidal trajectories. Multivariate multinomial logistic regression (for suicidal ideation trajectories, using Trajectory 1 as the reference category) and logistic regression (for suicide attempt trajectories, using Trajectory 1 as the reference category) were used to predict the class membership of the identified trajectories. Four steps were conducted successively: (1) Model 1 examined social network structures (i.e. family structure, household size, peer size, and peer density); (2) Model 2 examined social network functions (i.e. family cohesion, peer support, school connectedness, and neighborhood support); (3) Model 3 examined all social network variables. Models 1–3 controlled for sociodemographic characteristics and depression. Model 4 included a series of interaction terms (social networks × sociodemographic variables) to investigate the role of different social identities in the associations between social networks and suicidal trajectories.

## Results

### Descriptive statistics

Sociodemographic characteristics are presented in Table 1. The mean age at Wave I was 15.3 years (s.d. = 1.61). The sample was relatively evenly distributed by sex (54.58% female). Most respondents were heterosexual (86.08%), and non-Hispanic White (56.49%), had a mother whose highest educational level was lower than high school (57.27%). Nearly a third lived in a family received public assistance (26.96%). The prevalence of suicidal ideation decreased from baseline (13.64%) to 11.16% (Wave II), 6.47% (Wave III), and increased to 7.28% (Wave IV). The prevalence of suicide attempts continued to decrease from Wave I (3.91%), Wave II (3.40%), Wave III (1.69%), to Wave IV (1.45%). Adolescents with baseline suicide attempts were significantly less likely to live with two married parents (3.78% *v.* 96.22%, *p* < 0.05) and reported lower scores in family cohesion, peer support, school connectedness, and neighborhood support (*p* < 0.001).

**Table 1.** Sample characteristics and group comparisons by suicidal behaviors^a^

	Total	Suicidal ideation at W1	Non-suicidal ideation at W1	*p*^b^	Suicide attempts at W1	Non-suicide attempts at W1	*p*^b^
	(*n* = 9421)	(*n* = 1276, 13.64%)	(*n* = 8078, 86.36%)		(*n* = 366, 3.91%)	(*n* = 8986, 96.09%)	
Sociodemographic characteristics							
Age at baseline (M ± s.d.)	15.30 (1.61)	**15.43 (1.53)**	**15.24 (1.62)**	**<0.001**	15.26 (1.52)	15.26 (1.62)	0.956
Sex, *n*(%)							**<0.001**
Male	4279 (45.42)	**412 (32.29)**	**3830 (47.41)**		**85 (23.22)**	**4156 (46.25)**	
Female	5142 (54.58)	**864 (67.71)**	**4248 (52.59)**		**281 (76.78)**	**4830 (53.75)**	
Sexual identity, *n*(%)				**<0.001**			**<0.001**
Heterosexual	8101 (86.08)	**986 (77.27)**	**7037 (87.48)**		**258 (70.49)**	**7764 (86.73)**	
Sexual minority	1310 (13.92)	**290 (22.73)**	**1007 (12.52)**		**108 (29.51)**	**1188 (13.27)**	
Race/ethnicity, *n*(%)				**0.011**			0.526
White	5319 (56.49)	**732 (57.37)**	**4564 (56.52)**		197 (53.83)	5098 (56.75)	
Black	1931 (20.51)	**218 (17.08)**	**1698 (21.03)**		74 (20.22)	1841 (20.49)	
Hispanic	1448 (15.38)	**210 (16.46)**	**1221 (15.12)**		68 (18.58)	1363 (15.17)	
Asian	597 (6.34)	**97 (7.60)**	**491 (6.08)**		23 (6.28)	565 (6.29)	
American Indian	73 (0.78)	**14 (1.10)**	**58 (0.72)**		3 (0.82)	69 (0.77)	
Other races	35 (0.37)	**3 (0.24)**	**32 (0.40)**		0 (0.00)	35 (0.39)	
Multiracial	13 (0.14)	**2 (0.16)**	**11 (0.14)**		1 (0.27)	12 (0.13)	
Maternal education, *n*(%)				**<0.001**			**0.031**
No school/less than high school	1405 (16.44)	**229 (19.72)**	**1176 (15.92)**		**69 (20.72)**	**1336 (16.27)**	
High school or equivalent	3490 (40.83)	**418 (36.00)**	**3072 (41.59)**		**131 (39.34)**	**3359 (40.90)**	
Some college	1109 (12.98)	**163 (14.04)**	**946 (12.81)**		**52 (15.62)**	**1057 (12.87)**	
Graduate school and higher	2543 (29.75)	**351 (30.23)**	**2192 (29.68)**		**81 (24.32)**	**2461 (29.96)**	
Public assistance, *n*(%)				**0.002**			**0.002**
None	6089 (73.04)	**770 (69.24)**	**5280 (73.67)**		**214 (65.24)**	**5835 (73.40)**	
At least one	2247 (26.96)	**342 (30.76)**	**1887 (26.33)**		**114 (34.76)**	**2115 (26.60)**	
*Social networks*							
Network structure							
Family structure, *n*(%)	6096 (72.92)	796 (12.97)	5259 (87.03)	0.134	**218 (3.78)**	**5835 (96.22)**	**0.024**
Household size, *n*(%)	7466 (77.04)	991 (13.57)	6429 (86.43)	0.895	281 (4.05)	7139 (95.95)	0.853
Peer network size, M ± s.d.	4.73 (3.68)	4.70 (3.62)	4.89 (3.70)	0.951	5.17 (3.42)	4.87 (3.70)	0.767
Peer network density, M ± s.d.	0.29 (0.14)	0.28 (0.14)	0.29 (0.14)	0.173	0.28 (0.13)	0.29 (0.14)	0.087
Network function							
Family cohesion, M ± s.d.	3.77 (0.82)	**3.25 (0.88)**	**3.86 (0.78)**	**<0.001**	**3.16 (0.91)**	**3.80 (0.81)**	**<0.001**
Peer support, M ± s.d.	3.93 (0.66)	**3.73 (0.74)**	**3.97 (0.64)**	**<0.001**	**3.68 (0.75)**	**3.95 (0.65)**	**<0.001**
School connectedness, M ± s.d.	3.48 (0.54)	**3.23 (0.58)**	**3.54 (0.52)**	**<0.001**	**3.15 (0.63)**	**3.52 (0.53)**	**<0.001**
Neighborhood support, M ± s.d.	0.81 (0.26)	**0.78 (0.28)**	**0.82 (0.26)**	**<0.001**	**0.81 (0.27)**	**0.82 (0.26)**	**0.011**
Depression (M ± s.d.)	0.66 (0.47)	**1.07 (0.57)**	**0.56 (0.42)**	**<0.001**	**1.18 (0.61)**	**0.61 (0.45)**	**<0.001**

In Table 1, the left side presents the sociodemographic and depressive symptoms of the total sample based on unimputed data. The right side shows the bivariate analysis between suicidal behaviors and sociodemographic characteristics as well as depression. Cells in bold print indicate significant results.

a Participant numbers based on unweighted data and percentages based on weighted data.

b Comparing participants with suicidal ideation and suicide attempts to those without suicide attempts. χ*^2^* analyses were used for categorical variables, whereas the *t* test was used for continuous variables.

### Suicidal trajectories

Online Supplementary Table A1 displays fit statistics for LCGA models. The three-class solution was optimal for suicidal ideation trajectories, and the two-class solution was optimal for suicide attempt trajectories. For suicidal ideation trajectories, the adjusted BIC values were smallest among the three- and four-class solutions, but the four-class solution was excluded due to the small size of one additional class (0.01%) and the nonsignificant LRT statistic. The entropy of the three-class solution (0.845) also exceeded the criterion for good class separation (⩾0.60). For suicide attempt trajectories, the two-class solution had the lowest adjusted BIC of all solutions, had good class separation (entropy = 0.869), and a generalizable proportion of the sample in each class. Examining theoretical cohesiveness supported class selection.

**Fig. 1.**
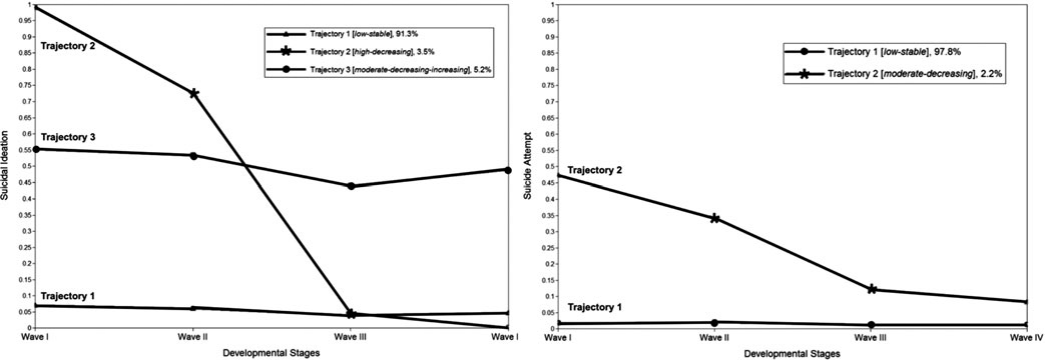
Trajectories of suicidal ideation and suicide attempts.

Figure 1 displays the three suicidal ideation trajectories. Most of the analytic sample was classified into Trajectory 1 (*low-stable*, 91.32%), indicating a consistently low likelihood of suicidal ideation from adolescence to adulthood. Trajectory 2 (*high-decreasing*, 3.45%) consisted of adolescents with a high initial suicidal ideation that decreased over time. Trajectory 3 (*moderate-decreasing-increasing*, 5.23%) represented adolescents reporting a moderate likelihood of suicidal ideation during adolescence, which decreased while transitioning to emerging adulthood but increased in young adulthood. For suicide attempt trajectories (Fig. 1), most participants were classified into Trajectory 1 (*low-stable*, 97.79%), which showed a persistently low likelihood of attempting suicide. Trajectory 2 (*moderate-decreasing*, 2.21%) contained adolescents with moderate initial suicide attempts, which decreased later. Moderate indicates in the middle (not high or low) of risks of suicidal ideation and suicide attempts, compared with other suicidal trajectories.

### Social networks during adolescence and suicidal trajectories

Bivariate analyses indicated significant differences in family cohesion, peer support, school connectedness, and neighborhood support, but not family and peer network structures across suicidal trajectories (online Supplementary Table A2). The bivariate results were consistent with the previous empirical studies (Bearman & Moody, 2004; Cha et al., 2018a). Table 2 presents the unique contributions of each predictor in explaining suicidal trajectories. For suicidal ideation, adolescents with higher family cohesion were less likely to be in Trajectory 2 [*high-decreasing*; odds ratio (OR) 0.52, 95% confidence interval (CI) 0.41–0.66] and Trajectory 3 (*moderate-decreasing-increasing*; OR 0.63, 95% CI 0.52–0.77) when including family cohesion, peer support, school connectedness, and neighborhood support only (Model 2). After accounting for all network variables (Model 3), greater family cohesion remained a unique predictor of engaging in *high-decreasing* (OR 0.54, 95% CI 0.40–0.73) and *moderate-decreasing-increasing* (OR 0.74, 95% CI 0.59–0.91) suicidal ideation trajectories. Higher family cohesion also lowered the likelihood of belonging to suicide attempt Trajectory 2 (*moderate-decreasing*; OR 0.66, 95% CI 0.45–0.96).

**Table 2.** Results of the multinomial logistic regression^a^

	SI Trajectory 2 *v*. Trajectory 1			SI Trajectory 3 *v*. Trajectory 1			SA Trajectory 2 *v*. Trajectory 1		
	Model 1: structure	Model 2: function	Model 3: structure + function	Model 1: structure	Model 2: function	Model 3: structure + function	Model 1: structure	Model 2: function	Model 3: structure + function
	OR (95% CI)	OR (95% CI)	OR (95% CI)	OR (95% CI)	OR (95% CI)	OR (95% CI)	OR (95% CI)	OR (95% CI)	OR (95% CI)
Sex									
Male	Ref.	Ref.	Ref.	Ref.	Ref.	Ref.	Ref.	Ref.	Ref.
Female	1.82** (1.16–2.86)	1.48* (1.01–2.18)	1.89** (1.22–2.92)	0.90 (0.55**–**1.47)	0.86 (0.58**–**1.27)	0.92 (0.56**–**1.53)	0.63 (0.34**–**1.15)	0.49** (0.31–0.77)	0.66 (0.37**–**1.17)
Sexual identity									
Heterosexual	Ref.	Ref.	Ref.	Ref.	Ref.	Ref.	Ref.	Ref.	Ref.
Sexual minority	1.90* (1.09–3.32)	1.82** (1.22–2.72)	1.72 (1.00**–**2.97)	3.18*** (2.08–4.88)	2.84*** (1.97–4.08)	3.07*** (2.00–4.73)	2.94*** (1.73–5.00)	2.61*** (1.69–4.02)	2.81*** (1.65–4.78)
Age (Wave 1)	1.05 (0.93**–**1.19)	1.01 (0.91**–**1.12)	1.01 (0.89**–**1.15)	0.99 (0.90**–**1.10)	0.92 (0.85**–**1.01)	0.97 (0.87**–**1.07)	0.99 (0.86**–**1.14)	0.92 (0.81**–**1.05)	0.97 (0.84**–**1.11)
Race/ethnicity									
White	Ref.	Ref.	Ref.	Ref.	Ref.	Ref.	Ref.	Ref.	Ref.
Black	0.35*** (0.19–0.63)	0.48** (0.29–0.80)	0.36** (0.19–0.67)	0.45** (0.27–0.77)	0.56** (0.37–0.83)	0.47** (0.27–0.80)	0.42 (0.15**–**1.16)	0.55 (0.26**–**1.20)	0.44 (0.16**–**1.21)
Hispanic	0.52 (0.26**–**1.06)	0.99 (0.54**–**1.79)	0.46* (0.22–0.96)	0.67 (0.41**–**1.09)	0.79 (0.53**–**1.17)	0.66 (0.40**–**1.09)	0.83 (0.44**–**1.58)	0.90 (0.53**–**1.53)	0.82 (0.43**–**1.58)
Asian	1.18 (0.33**–**4.19)	1.00 (0.38**–**2.66)	1.14 (0.32**–**4.01)	0.62 (0.27**–**1.45)	0.61 (0.30**–**1.26)	0.66 (0.30**–**1.47)	0.18* (0.05–0.70)	0.33 (0.07**–**1.49)	0.18* (0.05–0.73)
All other race	0.18* (0.04–0.92)	0.13** (0.04–0.45)	0.17 (0.03**–**1.05)	0.85 (0.34**–**2.14)	0.59 (0.24**–**1.43)	0.80 (0.32**–**2.00)	0.43 (0.11**–**1.68)	0.51 (0.18**–**1.46)	0.44 (0.10**–**2.00)
Maternal education									
No school/less than high school	Ref.	Ref.	Ref.	Ref.	Ref.	Ref.	Ref.	Ref.	Ref.
High school or equivalent	0.97 (0.62**–**1.53)	0.85 (0.54**–**1.36)	0.94 (0.59**–**1.49)	1.12 (0.69**–**1.83)	1.32 (0.89**–**1.97)	1.09 (0.67**–**1.76)	1.16 (0.57**–**2.39)	1.59 (0.95**–**2.67)	1.14 (0.54**–**2.39)
Some college	0.87 (0.39**–**1.94)	0.9 (0.45**–**1.78)	0.78 (0.36**–**1.70)	1.56 (0.90**–**2.73)	1.39 (0.82**–**2.35)	1.47 (0.84**–**2.57)	1.45 (0.60**–**3.51)	1.63 (0.82**–**3.24)	1.37 (0.55**–**3.41)
Graduate school and higher	1.19 (0.67**–**2.13)	1.28 (0.73**–**2.25)	1.12 (0.61**–**2.05)	0.95 (0.57**–**1.61)	1.43 (0.91**–**2.26)	0.93 (0.56**–**1.55)	1.40 (0.70**–**2.83)	1.59 (0.89**–**2.86)	1.31 (0.64**–**2.66)
Public assistance									
None	Ref.	Ref.	Ref.	Ref.	Ref.	Ref.	Ref.	Ref.	Ref.
At least one	0.89 (0.55**–**1.44)	0.82 (0.56**–**1.22)	0.84 (0.52**–**1.37)	0.77 (0.53**–**1.12)	1.01 (0.75**–**1.36)	0.76 (0.52**–**1.09)	1.42 (0.78**–**2.59)	1.72* (1.02–2.90)	1.35 (0.72**–**2.51)
*Social networks*									
Network structure^b.^									
Family structure	1.28 (0.72**–**2.25)		1.39 (0.77**–**2.51)	0.88 (0.58**–**1.35)		0.88 (0.57**–**1.35)	0.77 (0.38**–**1.58)		0.77 (0.38**–**1.56)
Household size	1.13 (0.66**–**1.93)		1.1 (0.64**–**1.92)	1.25 (0.82**–**1.90)		1.26 (0.82**–**1.93)	1.59 (0.85**–**2.97)		1.54 (0.82**–**2.90)
Peer network size	1.02 (0.96**–**1.08)		1.03 (0.97**–**1.09)	0.98 (0.94**–**1.03)		0.99 (0.95**–**1.03)	1.02 (0.95**–**1.08)		1.02 (0.96**–**1.09)
Peer network density	1.00 (0.27**–**3.66)		1.03 (0.27**–**3.96)	1.69 (0.68**–**4.20)		1.71 (0.69**–**4.26)	0.45 (0.09**–**2.33)		0.42 (0.08**–**2.07)
Network function									
Family cohesion		0.52*** (0.41–0.66)	0.54*** (0.40–0.73)		0.63*** (0.52–0.77)	0.74** (0.59–0.91)		0.66* (0.45–0.96)	0.70 (0.46**–**1.06)
Peer support		1.02 (0.80**–**1.30)	0.89 (0.66**–**1.18)		1.02 (0.80**–**1.30)	0.85 (0.63**–**1.14)		1.12 (0.76–1.65)	
School connectedness		0.77 (0.56–1.05)	0.82 (0.56–1.20)		0.77 (0.59–1.02)	0.83 (0.59–1.14)		0.90 (0.61–1.31)	1.16 (0.71–1.89)
Neighborhood support		0.87 (0.45–1.67)	0.70 (0.30–1.65)		1.28 (0.79–2.08)	1.71 (0.90–3.23)		0.87 (0.35–2.14)	1.52 (0.63–3.67)
Depression	7.78*** (5.61–10.80)	4.89*** (3.59–6.65)	4.84*** (3.32–7.05)	3.99*** (3.09–5.14)	2.91*** (2.25–3.75)	3.02*** (2.26–4.02)	7.81*** (5.55–11.00)	5.59*** (3.96–7.89)	6.05*** (4.11–8.91)

OR, odds ratio; 95% CI, 95% confidence interval; SI, suicidal ideation; SA, suicide attempts; Ref., reference group. Cells in bold print indicate significant results.

a Reference group = Trajectory 1.

b Family-level network structure included family structure (0 = all other arrangements; 1 = married, two parents) and household size (0 = ⩽ 3 people; 1 = ⩾ 4 people).

∗*p* < 0.05; ∗∗ *p* < 0.01; ∗∗∗ *p* ⩽0.001.

### Moderation effects of race/ethnicity, sex, and sexual identity

There were significant racial/ethnic differences in the association between social networks during adolescence and suicidal trajectories (Model 4, online Supplementary Fig. A1 and Table A3 in the online appendices). Black youth with high levels of family cohesion were less likely than their White counterparts to be in suicidal ideation Trajectory 3 (*moderate-decreasing-increasing*) than to Trajectory 1 (*low-stable*; OR 0.60, 95% CI 0.38–0.92). For Hispanic respondents, living in a household with more than four family members (OR 8.89, 95% CI 1.47–53.90) was significantly associated with a greater likelihood of being in suicidal ideation Trajectory 2 (*high-decreasing*) *versus* Trajectory 1 (*low-stable*). Hispanic participants were less likely to be in Trajectory 2 (*moderate-decreasing*) of suicide attempts when they perceived high school connectedness (OR 0.22, 95% CI 0.05–0.91).

Examining sex-specific differences (online Supplementary Fig. A2 and Table A4 in the online appendices) revealed that female respondents were less likely to be in suicidal ideation Trajectory 3 (*moderate-decreasing-increasing*) when living in a household with more than four family members (OR 0.44, 95% CI 0.19–0.89), while they were more likely than males to be in suicide attempt Trajectory 2 (*moderate-decreasing*) when having high levels of peer support (OR 2.91, 95% CI 1.19–7.13).

Online Supplementary Fig. A2 and Table A5 showed significant moderation effects of sexual identity. Sexual minorities were more likely to be in Trajectory 2 (*high-decreasing*) when having a densely connected peer network (OR 1.19, 95% CI 1.02–1.40) and low levels of neighborhood support (OR 0.10, 95% CI 0.02–0.57).

## Discussion

### Significance

This study addresses a gap in knowledge about the link between social networks during early adolescence and the changing patterns of suicidal behaviors across developmental stages (i.e. suicidal trajectories). Using a longitudinal and nationally representative sample, we identified three distinct trajectories of suicidal ideation (*low-stable*, *high-decreasing*, *moderate-decreasing-increasing*) and two suicide attempt trajectories (*low-stable*, *moderate-decreasing*) over 14 years. Guided by the NEM, we examined the associations between social networks during adolescence and different suicidal trajectories. Across all social network characteristics, higher-level family cohesion was significantly associated with lower probabilities of engaging in *high-decreasing* and *moderate-decreasing-increasing* suicidal ideation trajectories, as well as *moderate-decreasing* suicide attempt trajectories. Social network influences further differed across race/ethnicity, sex, and sexual identity. Our findings were among the few longitudinal studies to strengthen the evidence that network-based interventions are key to prevent suicidal behaviors over time and should be a key component in prevention programming.

### Suicidal trajectories

Suicidal ideation trajectories identified in this study were consistent with previous research (Nkansah-Amankra, 2013; Rueter et al., 2008). Females, sexual minorities, and individuals living in low SES families were more likely to be in Trajectories 2 (*high-decreasing*) and 3 (*moderate-decreasing-increasing*). This aligns with previous literature that adolescents in these sociodemographic subgroups had greater exposure to adverse environmental changes and exhibited comorbid problem behaviors (Cha et al., 2018a), which in turn increased their higher likelihood of suicidal ideation in the early stages of life course (Cha et al., 2018a; Fazel & Runeson, 2020; Franklin et al., 2017). Sexual minorities were more represented in Trajectory 3 (*moderate-decreasing-increasing*), which is consistent with the strong association between discrimination and suicidal behaviors among sexual minorities when transitioning from adolescence to adulthood (Giano et al., 2020; Ream, 2019).

Suicide attempt trajectories found in this study were inconsistent with previous findings identifying one trajectory or three trajectories (Thompson & Swartout, 2018), which may be because of the different covariates adjusted in the conditional LGCA. The results that sexual minorities and those living in low-income families were more likely to be in suicide attempt Trajectory 2 (*moderate-decreasing*) were consistent with prior research and supported our class selection (Cha et al., 2018b; Ream, 2019). More research is needed to identify critical periods and at-risk sociodemographic subpopulations across suicide attempt trajectories.

### Social networks and suicidal ideation trajectories

This study provides significant evidence supporting that improving family cohesion is a promising strategy for reducing the likelihood of engaging in suicidal behaviors from adolescence through young adulthood. The importance of family cohesion was consistent with previous research revealing its effect on future suicidal behaviors (Fergusson et al., 2000). Compared to social supports from peers, schools, and communities, family cohesion was found to be a stronger predictor of suicidal ideation and suicide attempts (Kerr et al., 2006; Lindsey, Joe, & Nebbitt, 2010; Miller et al., 2015). Our results strengthen the existing literature by demonstrating the protective effect of family cohesion in high-risk suicidal trajectories.

### Sociodemographic differences

For Black participants, greater family cohesion protected against high adolescent suicidal ideation and increasing risk when transitioning to early adulthood. Family matters for Black adolescents in terms of increasing their help-seeking behaviors and social support (Lindsey et al., 2010). This study extended the previous literature by addressing the pivotal role of family cohesion in sustainably reducing the risks of suicidal ideation among Black adolescents and adults. The protective effect of family, however, was not observed among Hispanic respondents. Cultural values such as familism (i.e. family-centered values) or support from other social contexts may provide more explanatory power of suicidal trajectories among the Hispanic population (Chu, Goldblum, Floyd, & Bongar, 2010; Xiao, Romanelli, Vélez-Grau, & Lindsey, 2020). Given the dearth of related literature, more studies are needed to further understand the role of families among this population.

Female participants were less likely to exhibit a *moderate-decreasing-increasing* suicidal ideation trajectory when living with more than four family members. This mirrors previous results suggesting denser family networks as an important protective factor against suicidal ideation for females, but not for males (Kerr et al., 2006). The greater likelihood of females belonging to higher-risk suicidal trajectories was consistent with previous findings suggesting gender-specific associations in friendship and suicidal behaviors; for example, females having greater risks of suicidal ideation when socially isolated and having less connected peer networks (Bearman & Moody, 2004; Kerr et al., 2006). Strong connections with close friends may increase the spread of sensitive information and health risk behaviors (e.g. suicide contagion) among females over time (Prinstein, Boergers, Spirito, Little, & Grapentine, 2000).

Sexual minorities reported different suicidal trajectories when they received different levels of support from non-family members (e.g. improving peer support and neighborhood support). Since sexual minority populations report more family rejection (Giano et al., 2020), they were more likely than their heterosexual counterparts to seek help from adults unrelated to them, which may explain their lower probability of belonging to *moderate-decreasing-increasing* suicidal ideation trajectory when reporting higher-levels of neighborhood support (Cha et al., 2018a). Future research shall examine the mechanisms linking to the changes in suicidal behaviors among sexual minorities.

### Implications

This study is one of the first to demonstrate the importance of social networks across socioecological contexts (i.e. families, peers, neighborhoods) in predicting suicidal trajectories over the life course. Future research shall investigate the underlying mechanisms and develop family-engaged and culturally adaptive interventions to prevent early-onset and relapse of suicidal behaviors among adolescents transitioning to adulthood. Further information on network content (i.e. what information has been exchanged) shall be considered (Pescosolido, 1991, 1992; Wray et al., 2011). More longitudinal studies across longer periods are needed to examine the causal mechanisms through which multidimensional social networks influence suicidal trajectories.

The World Health Organization (2012) addresses the importance of developing a national suicide prevention strategy through public health and multi-sectoral stepwise approaches. This study strengthens the strategy by providing evidence that social networks across the socioecological sources of early adolescent life could affect the suicidal trajectories over the life course. Policymakers shall engage collective efforts to build up supportive communities, encourage help-seeking behaviors, reduce stigma and discrimination, and provide timely services for vulnerable populations. For racial/ethnic and sexual minorities, it is important to allocate accessible and affordable mental health resources for screening, counseling, and treatment. Health promotion programs should target de-stigmatization and building supportive networks.

Clinically, our results highlight the potential of network-informed interventions to improve the effectiveness of youth suicide prevention. For example, by improving family support and psychoeducation, the Youth-Nominated Support Team-Version II intervention was found to be more cost-effective and efficient than traditional intervention (Glenn et al., 2015). With the rapid development of information and telehealth tools since the COVID-19 pandemic, clinical research and interventions could be scaled up by utilizing different sources of communication, assessing social network data (e.g. social media, texting, or email) and designing telepsychiatry-based interventions (Valente, 2012). Public health professionals and social work practitioners shall also implement tailored interventions that serve the needs of individuals from diverse sociodemographic backgrounds (Cha et al., 2018b; Chu et al., 2010; Lindsey et al., 2019; Xiao, Wong, Cheng, & Yip, 2021).

### Limitations

Despite the significance and rigorous design of this study, some limitations should be noted. First, data collected in Add Health are self-reported and may contain measurement bias. Prior studies, however, have established the measurement reliability and validity, which were further improved by using the CAPI/ACASI technologies (Sieving et al., 2001). Second, suicidal ideation and suicide attempts were binary indicators, without accounting for the frequency and severity. Third, measures of social networks, while consistent with previous theoretical and empirical studies, are not standardized scales, and some showed relatively low internal consistency. Future studies shall use validated scales, including the Multidimensional Scale of Perceived Social Support (Zimet, Dahlem, Zimet, & Farley, 1988). Fourth, we included standard race/ethnic categories, which may mask race/ethnic heterogeneity within these groups (e.g. Hispanic/Latino adolescents who also identified as White/Black). Fifth, the data in the last three waves were assessed across 5 or more years. Suicidal behaviors occurred within shorter time intervals may be underreported. Sixth, due to the unavailability of the relevant construct, this study did not assess network content (e.g. beliefs, cultural values, and attitudes) in NEM to capture the quality and substance of social networks (Pescosolido, 1991). Future studies should examine all three aspects of social network influences as conceptualized by Pescosolido (1991). Seventh, given the negative impact of adverse childhood experiences (e.g. sexual abuse, stressful childhood events) on suicidal behaviors and mental disorders (Jenkins et al., 2005; Lu & Xiao, 2019), social network influences on suicidal trajectories may differ between those who experienced childhood trauma and those who did not, as indicated by the Stress-Diathesis Theory (Mann & Rizk, 2020; Oquendo et al., 2014). Future studies that measure adverse childhood experiences are encouraged to further explore potential subgroup differences. Besides, future studies should investigate possible differences in suicidal trajectories across other family structures (e.g. presence of grandparents in three-generation families) during adolescent development. Further investigation of the significant role of family cohesion in protecting individuals from suicide risks should also be conducted to strengthen the practical evidence. Eighth, we did not include other underlying conditions of suicide (e.g. minority stress, cultural values) and multilevel risks across schools (e.g. school attachment) and community (e.g. rural/urban) contexts. Finally, Add Health collected data among students attending school. Adolescents who dropped out of high school, were homeless, or were runaways had greater suicide risks, but were not included in the baseline sample (Cha et al., 2018a).

## Conclusion

Given the increasing trends and disparities in suicidal behaviors across sociodemographic groups and the ripple effect it has on individual wellbeing, family functioning, and community capacity (Cha et al., 2018a; Nock et al., 2019), it is important to identify the underlying factors affecting suicidal ideation and suicide attempts over time. This study contributes to the literature by addressing health disparities across race/ethnicity, sex, sexual identity, and SES in the association between social networks in early adolescence and suicidal trajectories. Family cohesion was the most consistent social network factor during adolescence that influenced the likelihood of engaging in higher-risk trajectories. Findings from this study have meaningful implications for research, policies, and clinical practices. Engaging families and improving family cohesion may be a promising next step in preventing early-stage risks of suicidal ideation and suicide attempts, as well as the surge of risk when transitioning from emerging adulthood to young adulthood. Researchers, policymakers, and clinicians should develop new, more nuanced research designs to address the ‘social’ factor in the biopsychosocial model in suicide research and develop tailored suicide prevention and intervention for racial minorities, females, and sexual minorities.

## Supplementary material

For supplementary material accompanying this paper visit https://doi.org/10.1017/S0033291721000465. click here to view supplementary material
