# Supplementary material for: Adolescent social networks matter for suicidal trajectories: disparities across race/ethnicity, sex, sexual identity, and socioeconomic status
Source: Psychol Med. 2021 Mar 3;52(15):3677–88. doi: 10.1017/S0033291721000465 (PMC9772914; doi:10.1017/S0033291721000465)
Supplement: Supplementary file 1 [file S0033291721000465sup.zip › S0033291721000465sup001.docx]

# Figure A1

###
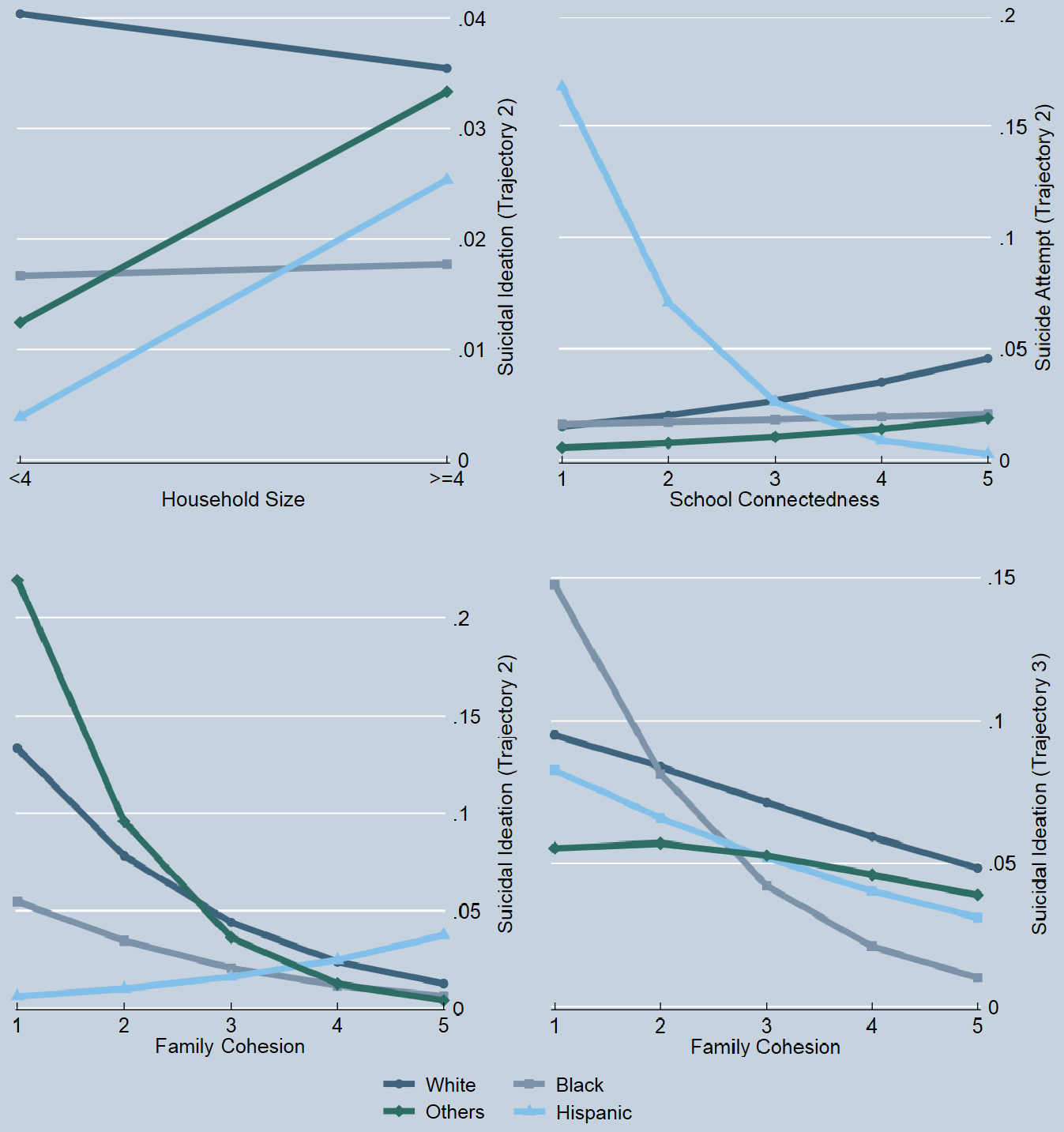
Moderation Effect of Race/ethnicity on the Association between Social Networks and Suicidal Trajectories

# Figure A2

### Moderation Effect of Sex and Sexual Identity on the Association between Social Networks and Suicidal Trajectories


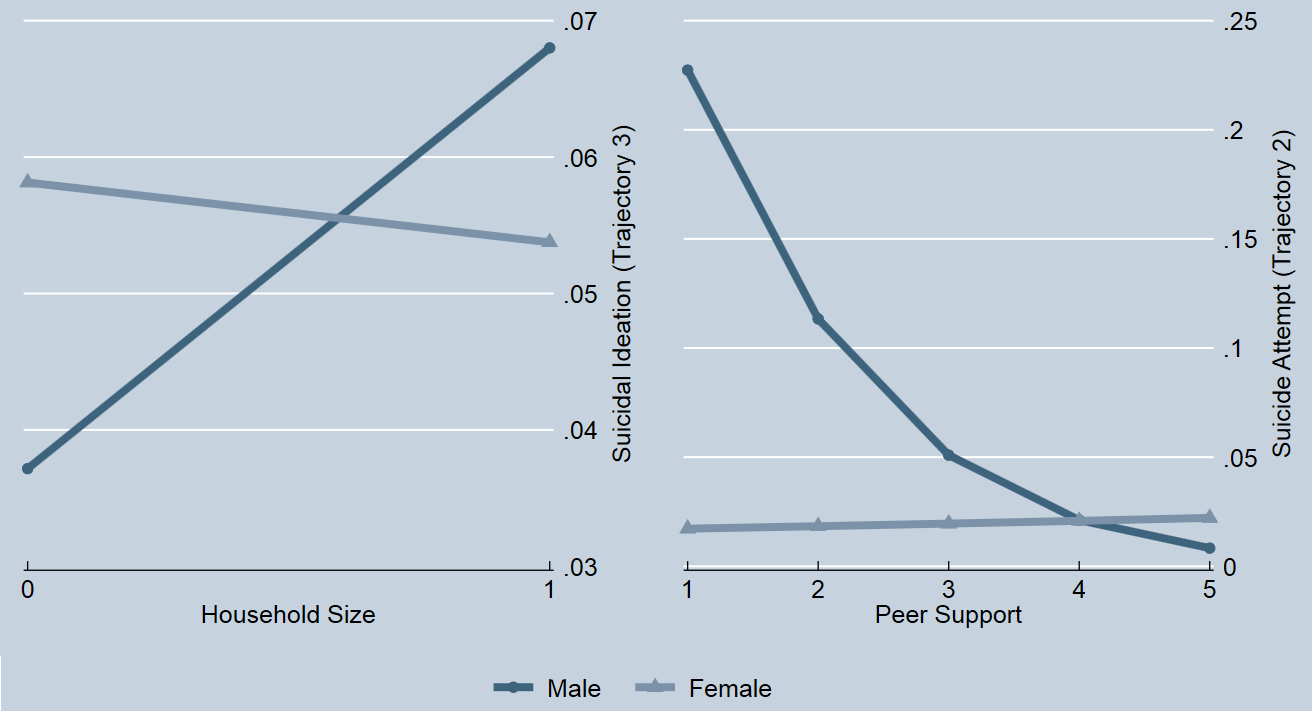

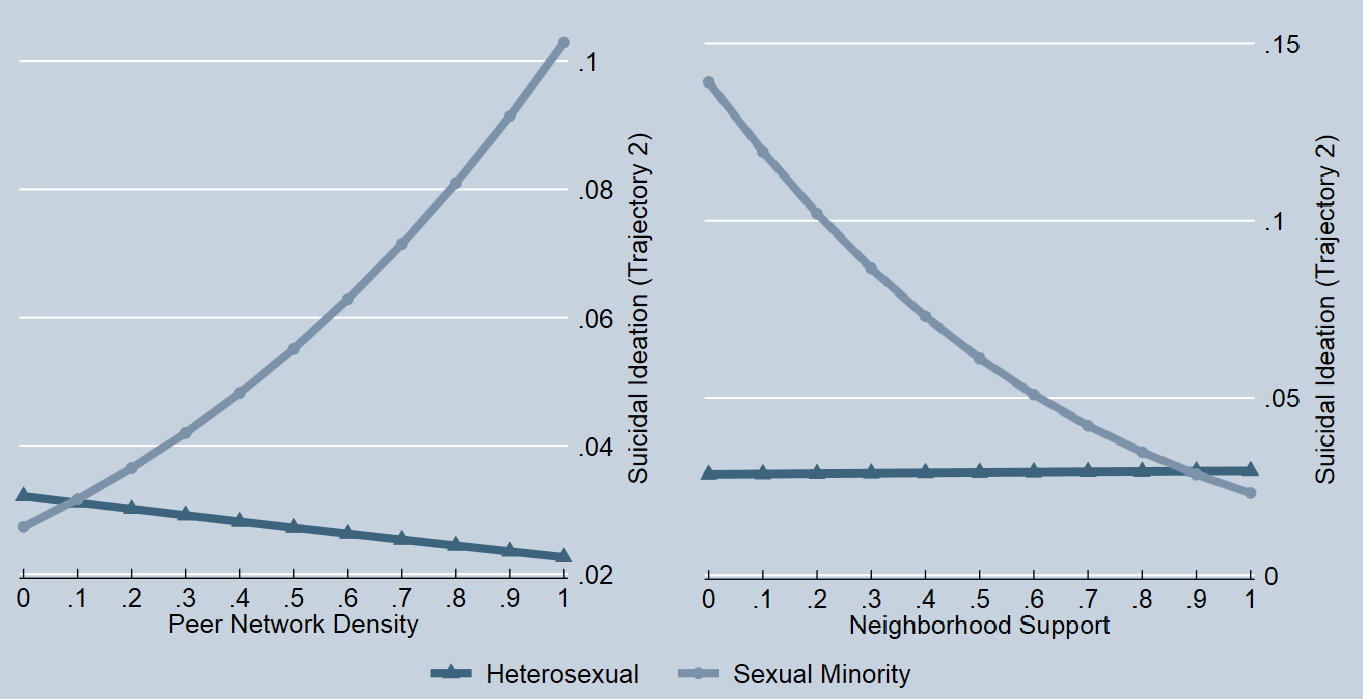


Table A1

*Summary of Latent Class Growth Analysis Model Identification and Fit Statistics*

| No. of Classes | AIC | BIC | Adjusted BIC | Smallest Class, % | Entropy | LMR-LRT | *P* |
| --- | --- | --- | --- | --- | --- | --- | --- |
| *Suicidal Ideation* |  |  |  |  |  |  |  |
| 1 | 24147.4 | 24333.3 | 24250.7 | **-** | **-** | N/A | N/A |
| 2 | 22966.8 | 23174.1 | 23082.0 | 8.95% | 0.713 | 1156.054 | 0.00 |
| **3** | **22892.2** | **23121.1** | **23019.4** | **3.42%** | **0.846** | **76.848** | **0.13** |
| 4 | 22870.5 | 23120.8 | 23009.5 | **0.83%** | **0.881** | 27.355 | 0.05 |
| 5 | 22875.9 | 23147.6 | 23026.9 | 0.00% | 0.800 | 0.209 | 0.45 |
| *Suicide Attempt* |  |  |  |  |  |  |  |
| 1 | 9254.1 | 9440.0 | 9357.4 | **-** | **-** | N/A | N/A |
| **2** | **8939.3** | **9146.6** | **9054.5** | **2.21%** | **0.816** | **306.95** | **0.00** |
| 3 | 8935.7 | 9164.6 | 9062.9 | 0.71% | 0.870 | 8.99 | 0.49 |
| 4 | 8939.6 | 9189.9 | 9078.7 | 0.06% | 0.900 | 2.05 | 0.60 |
| 5 | 8945.4 | 9217.1 | 9096.4 | 0.04% | 0.902 | 0.21 | 0.58 |

*Note.* AIC = Akaike Information Criterion; BIC = Bayesian Information Criteria; Adjusted BIC = Sample size adjusted Bayesian Information Criteria; LMR-LRT = Lo-Mendell-Rubin adjusted likelihood ratio test. Bolded models were selected.

Table A2

*Sociodemographic Characteristics, Social Networks and Psychological Differences across Suicidal Trajectories^a^*

|  | ***Suicidal Ideation Trajectory*** | | | | | ***Suicide Attempt Trajectory*** | | | |
| --- | --- | --- | --- | --- | --- | --- | --- | --- | --- |
|  | **Trajectory 1** | **Trajectory 2** | **Trajectory 3** | ***p*** | ***χ² / F*** | **Trajectory 1** | **Trajectory 2** | ***p*** | ***χ² / t*** |
|  | **[*Low-stable*]** | **[*High-decreasing*]** | **[*Moderate-decreasing-increasing*]** |  |  | **[*Low-stable*]** | **[*Moderate-decreasing*]** |  |  |
|  | **[*n* = 8,603, 91.32%]** | **[*n* = 325, 3.45%]** | **[*n* = 493, 5.23%]** |  |  | **[*n* = 9,213, 97.79%]** | **[*n* = 208, 2.21%]** |  |  |
| **Age [*M*±*SD*]** | 15.02 [1.62] | 15.28 [1.47] | 15.01 [1.63] | 0.120 | *F*[2, 9418] = 2.12 | 15.04 [1.61] | 14.89 [1.53] | 0.173 | *t* = 1.36 |
| **Sex, n [%]** |  |  |  | **<0.001** | ***χ*²[2] = 55.59** |  |  | 0.619 | *χ*²[1] = 0.25 |
| Male | **4007 [46.58]** | **98 [30.15]** | **174 [35.29]** |  |  | 4181 [45.38] | 98 [47.12] |  |  |
| Female | **4596 [53.42]** | **227 [69.85]** | **319 [64.71]** |  |  | 5032 [54.62] | 110 [52.88] |  |  |
| **Sexual Identity, *n*[%]** |  |  |  | **<0.001** | ***χ*²[2] = 195.99** |  |  | **<0.001** | ***χ²*[1] = 30.01** |
| Heterosexual | **7532 [87.55]** | **246 [75.69]** | **330 [66.94]** |  |  | **7956 [86.36]** | **152 [73.08]** |  |  |
| Sexual minority | **1071 [12.45]** | **79 [24.31]** | **163 [33.06]** |  |  | **1257 [13.64]** | **56 [26.92]** |  |  |
| **Race/ethnicity, *n*[%]** |  |  |  | 0.099 | *χ*²[12] = 18.61 |  |  | 0.154 | *χ*²[6] = 9.37 |
| White | 4833 [56.18] | 192 [59.08] | 295 [56.47] |  |  | 5205 [56.50] | 115 [55.29] |  |  |
| Black | 1804 [20.97] | 47 [14.46] | 83 [16.84] |  |  | 1901 [20.63] | 33 [15.87] |  |  |
| Hispanic | 1317 [15.31] | 58 [17.85] | 74 [15.01] |  |  | 1406 [15.26] | 43 [20.67] |  |  |
| Asian | 540 [6.28] | 25 [7.69] | 32 [6.49] |  |  | 585 [6.35] | 12 [5.77] |  |  |
| American Indian | 65 [0.76] | 2 [0.62] | 6 [1.22] |  |  | 70 [0.76] | 3 [1.44] |  |  |
| Other races | 33 [0.38] | 1 [0.31] | 1 [0.20] |  |  | 34 [0.37] | 1 [0.48] |  |  |
| Multiracial | 11 [0.13] | 0 [0.00] | 2 [0.41] |  |  | 12 [0.13] | 1 [0.48] |  |  |
| **Maternal Education, *n*[%]** |  |  |  | 0.061 | *χ*²[6] = 12.06 |  |  | 0.449 | *χ*²[3] = 2.65 |
| No school/Less than high school | 1369 [16.82] | 74 [23.79] | 71 [15.64] |  |  | 1568 [17.02] | 43 [20.67] |  |  |
| High school or equivalent | 3328 [40.90] | 112 [36.01] | 187 [41.19] |  |  | 3779 [41.02] | 83 [39.90] |  |  |
| Some college | 1067 [13.11] | 34 [10.93] | 61 [13.44] |  |  | 1183 [12.84] | 29 [13.94] |  |  |
| Graduate school and higher | 2373 [29.16] | 91 [29.26] | 135 [29.74] |  |  | 2683 [29.12] | 53 [25.48] |  |  |
| **Public assistance, *n*[%]** |  |  |  | 0.263 | *χ*²[2] = 2.67 |  |  | **0.042** | ***χ*²[1] = 4.13** |
| None | 5575 [73.18] | 209 [74.38] | 305 [69.79] |  |  | **6713 [72.86]** | **142 [68.27]** |  |  |
| At least one | 2043 [26.82] | 72 [25.62] | 132 [30.21] |  |  | **2500 [27.14]** | **66 [31.73]** |  |  |
| ***Social Networks*** |  |  |  |  |  |  |  |  |  |
| **Network Structure** *^b.^* |  |  |  |  |  |  |  |  |  |
| Family structure | 5573 [91.09] | 210 [3.39] | 313 [5.52] | 0.829 | *χ*²[2] = 0.58 | 5972 [97.79] | 124 [2.21] | 0.072 | *χ*²[1] = 9.19 |
| Household size | 6825 [90.82] | 261 [3.52] | 380 [5.66] | 0.492 | *χ*²[2] = 2.62 | 7307 [97.55] | 159 [2.45] | 0.927 | *χ*²[1] = 0.01 |
| Peer network size | 4.89 [3.68] | 5.07 [4.29] | 4.54 [3.28] | 0.135 | *F*[2, 6778] = 2.00 | 4.58 [3.69] | 4.79 [3.62] | 0.502 | *t* =-0.67 |
| Peer network density | 0.29 [0.14] | 0.28 [0.16] | 0.30 [0.14] | 0.758 | *F*[2, 6581] = 0.28 | 0.30 [0.14] | 0.27 [0.12] | 0.055 | *t* =1.92 |
| **Network Function** |  |  |  |  |  |  |  |  |  |
| Family cohesion | **3.82 [0.80]** | **3.01 [0.85]** | **3.39 [0.88]** | **<0.001** | ***F*[2, 9390] = 195.18** | **3.75 [0.81]** | **3.14 [0.96]** | **<0.001** | ***t* =10.61** |
| Peer support | **3.96 [0.65]** | **3.59 [0.73]** | **3.72 [0.71]** | **<0.001** | ***F*[2, 9412] = 48.73** | **3.93 [0.65]** | **3.67 [0.77]** | **<0.001** | ***t* =5.56** |
| School connectedness | **3.53 [0.53]** | **3.17 [0.57]** | **3.29 [0.59]** | **<0.001** | ***F*[2, 9393] = 90.33** | **3.48 [0.53]** | **3.15 [0.64]** | **<0.001** | ***t* =8.87** |
| Neighborhood support | **0.82 [0.26]** | **0.72 [0.30]** | **0.81 [0.27]** | **<0.001** | ***F*[2, 9389] = 16.10** | **0.81 [0.26]** | **0.77 [0.27]** | **0.039** | ***t* =2.06** |
| **Depression [*M*±*SD*]** | **0.60 [0.44]** | **1.18 [0.57]** | **0.93 [0.60]** | **<0.001** | ***F*[2, 9409] = 370.47** | **0.64 [0.46]** | **1.21 [0.63]** | **<0.001** | ***t* =-17.36** |

*Note.*

*^a^* Chi-square *(χ²)* tests for categorical variables, *Analysis of variance analyses (ANOVA)* for continuous variables by suicidal ideation trajectories, *t*-tests for continuous variables by suicide attempt trajectories. Cells in bold print indicate significant results.

*^b.^* Family-level network structure included family structure (0 = all other arrangements; 1 = married, two parents) and household size (0 = ≤ 3 people; 1 = ≥ 4 people).

Table A3

*Moderation Effect of Race/Ethnicity on Social Networks and Suicidal Trajectories^a^*

|  | **Suicidal Ideation** | | **Suicide Attempt** |
| --- | --- | --- | --- |
|  | **Trajectory 2**  **[*High-decreasing*]** | **Trajectory 3**  **[*Moderate-decreasing-increasing*]** | **Trajectory 2**  **[*Moderate-decreasing*]** |
|  | OR [95% CI] | OR [95% CI] | OR [95% CI] |
| **Sex** |  |  |  |
| Male |  |  |  |
| Female | 1.99** [1.28-3.09] | 0.93 [0.56-1.55] | 0.68 [0.39-1.20] |
| **Sexual Identity** |  |  |  |
| Heterosexual |  |  |  |
| Sexual minority | 1.80* [1.04-3.13] | 3.01*** [1.93-4.68] | 2.92*** [1.70-5.00] |
| **Age [Wave 1]** | 1.02 [0.90-1.16] | 0.96 [0.86-1.07] | 0.98 [0.85-1.13] |
| **Race/Ethnicity***^b.^* |  |  |  |
| White |  |  |  |
| Black | 0.20 [0.01-4.79] | 3.23 [0.11-98.30] | 0.00 [0.00-6.93] |
| Hispanic | 0.02 [0.00-6.31] | 1.1 [0.018-68.70] | 0.55 [0.02-19.50] |
| Others | 0.04 [0.00-23.9] | 19.4 [0.26-1455.60] | 0.69 [0.01-52.10] |
| **Maternal Education** |  |  |  |
| No school/Less than high school |  |  |  |
| High school or equivalent | 0.86 [0.54-1.37] | 1.13 [0.68-1.85] | 1.09 [0.51-2.34] |
| Some college | 0.71 [0.32-1.57] | 1.52 [0.86-2.69] | 1.27 [0.50-3.23] |
| Graduate school and higher | 1.07 [0.58-1.98] | 0.97 [0.58-1.62] | 1.24 [0.59-2.60] |
| **Public Assistance** |  |  |  |
| None |  |  |  |
| At least one | 0.85 [0.52-1.38] | 0.76 [0.52-1.11] | 1.31 [0.71-2.42] |
| ***Social Networks*** |  |  |  |
| **Network Structure** *^c^*^.^ |  |  |  |
| Family structure | 2.01* [1.00-4.05] | 1.05 [0.60-1.86] | 0.67 [0.31-1.44] |
| Household size | 0.87 [0.47-1.62] | 1.13 [0.69-1.85] | 1.48 [0.67-3.27] |
| Peer network size | 1.02 [0.96-1.09] | 0.99 [0.94-1.04] | 1.01 [0.93-1.10] |
| Peer network density | 0.48 [0.10-2.40] | 2.06 [0.75-5.62] | 0.35 [0.05-2.30] |
| **Network Function** |  |  |  |
| Family cohesion | **0.49*** [0.35-0.69]** | **0.78* [0.61-0.99]** | **0.61* [0.38-0.98]** |
| Peer support | 0.87 [0.62-1.23] | 0.84 [0.60-1.17] | 0.7 [0.40-1.24] |
| School detachment | 0.97 [0.64-1.47] | 0.8 [0.56-1.15] | 1.39 [0.78-2.47] |
| Neighborhood support | 0.58 [0.23-1.46] | 2.18 [0.99-4.79] | 1.44 [0.49-4.19] |
| **Race/Ethnicity × Network Structure** |  |  |  |
| Black × Married | 0.91 [0.25-3.33] | 0.66 [0.23-1.86] | 4.06 [0.76-21.8] |
| Hispanic × Married | **0.25 [0.06-1.00]** | 0.58 [0.21-1.59] | 1.11 [0.23-5.38] |
| Other race × Married | 0.14 [0.02-1.16] | 0.31 [0.08-1.31] | 0.18 [0.02-1.56] |
| Black × >=4 members | 1.28 [0.27-6.05] | 1.29 [0.41-4.04] | 0.46 [0.08-2.65] |
| Hispanic × >=4 members | **8.89* [1.47-53.90]** | 1.57 [0.34-7.33] | 18.00 [0.92-352.90] |
| Other race × >=4 members | 4.52 [0.25-81.1] | 1.59 [0.45-5.60] | 5.72 [0.50-66.1] |
| Black × Peer network size | 1.06 [0.86-1.31] | 1.04 [0.93-1.16] | 1.12 [0.95-1.32] |
| Hispanic × Peer network size | 1.02 [0.89-1.16] | 0.99 [0.86-1.15] | 1.03 [0.88-1.19] |
| Other race × Peer network size | 1.07 [0.86-1.35] | 1.00 [0.79-1.28] | 1 [0.85-1.19] |
| Black × Peer network density | 3.39 [0.09-123.30] | 1.06 [0.10-12.20] | 6.97 [0.25-197.00] |
| Hispanic × Peer network density | 2 [0.01-350.60] | 0.21 [0.00-10.20] | 0.42 [0.00-52.30] |
| Other race × Peer network density *^d.^* | **1409.5*** [28.9-68781.10]** | 0.12 [0.00-3.75] | 1.2 [0.04-40.00] |
| **Race/Ethnicity × Network Function** |  |  |  |
| Black × Family cohesion | 1.04 [0.49-2.21] | **0.60* [0.38-0.92]** | 2.51 [0.63-10.00] |
| Hispanic × Family cohesion | **3.25*** [1.72-6.17]** | 1.00 [0.53-1.88] | 1.49 [0.56-3.96] |
| Other race × Family cohesion | 0.55 [0.24-1.28] | 1.04 [0.45-2.40] | 1.3 [0.68-2.47] |
| Black × Peer support | 1.03 [0.49-2.16] | 0.99 [0.44-2.23] | 1.25 [0.48-3.26] |
| Hispanic × Peer support | 1.50 [0.38-5.89] | 0.81 [0.37-1.78] | 1.29 [0.49-3.37] |
| Other race × Peer support | 1.12 [0.43-2.89] | 1.24 [0.41-3.69] | 0.69 [0.25-1.95] |
| Black × School connectedness | 0.56 [0.15-2.01] | 1.33 [0.41-4.28] | 0.77 [0.25-2.41] |
| Hispanic × School connectedness | 0.48 [0.11-2.13] | 1.20 [0.42-3.45] | **0.22* [0.05-0.91]** |
| Other race × School connectedness | 0.90 [0.31-2.63] | 0.60 [0.17-2.08] | 1.04 [0.53-2.03] |
| Black × Neighborhood support | 7.23 [0.69-75.6] | 0.20 [0.031-1.29] | 2.51 [0.13-48.0] |
| Hispanic × Neighborhood support | 0.26 [0.01-4.94] | 1.28 [0.21-7.86] | 1.38 [0.07-28.3] |
| Other race × Neighborhood support | 12.4 [0.11-1463.80] | 0.13 [0.01-2.10] | 0.21 [0.03-1.41] |
| **Depression** | 5.14*** [3.50-7.56] | 3.02*** [2.25-4.03] | 6.33*** [4.22-9.48] |

*Note.* OR = odds ratio; 95% CI = 95% confidence interval. Cells in bold print indicate significant results for social networks. **p*<0.05; ***p*<0.01; ****p*<0.001

*^a.^* Reference group = Trajectory 1.

*^b^*^.^ Asian, Native American, multiracial and others were collapsed into one group due to small sample sizes.

*^c^*^.^ Family-level network structure included family structure (0 = all other arrangements; 1 = married, two parents) and household size (0 = ≤ 3 people; 1 = ≥ 4 people).

*^d.^* OR may not be reliable due to data sparseness.

Table A4

*Moderation Effect of Sex on Social Networks and Suicidal Trajectories^a^*

|  | **Suicidal Ideation** | | **Suicide Attempt** |
| --- | --- | --- | --- |
|  | **Trajectory 2**  **[*High-decreasing*]** | **Trajectory 3**  **[*Moderate-decreasing-increasing*]** | **Trajectory 2**  **[*Moderate-decreasing*]** |
|  | OR [95% CI] | OR [95% CI] | OR [95% CI] |
| **Sex** |  |  |  |
| Male |  |  |  |
| Female | 0.96 [0.06-14.50] | 1.23 [0.16-9.47] | 0.17 [0.00-3.95] |
| **Sexual Identity** |  |  |  |
| Heterosexual |  |  |  |
| Sexual minority | 1.66 [0.97-2.85] | 3.10*** [1.98-4.85] | 2.83*** [1.63-4.90] |
| **Age [Wave 1]** | 1.00 [0.88-1.14] | 0.96 [0.87-1.07] | 0.99 [0.86-1.14] |
| **Race/Ethnicity***^b.^* |  |  |  |
| White |  |  |  |
| Black | 0.37** [0.20-0.70] | 0.48** [0.28-0.81] | 0.41 [0.15-1.14] |
| Hispanic | 0.48 [0.23-1.02] | 0.66 [0.40-1.09] | 0.83 [0.43-1.58] |
| Others | 0.84 [0.30-2.34] | 0.72 [0.39-1.34] | 0.29* [0.09-0.91] |
| **Maternal Education** |  |  |  |
| No school/Less than high school |  |  |  |
| High school or equivalent | 0.95 [0.59-1.54] | 1.05 [0.66-1.69] | 1.19 [0.56-2.54] |
| Some college | 0.75 [0.35-1.61] | 1.39 [0.79-2.44] | 1.41 [0.56-3.54] |
| Graduate school and higher | 1.20 [0.65-2.20] | 0.92 [0.56-1.50] | 1.27 [0.63-2.58] |
| **Public Assistance** |  |  |  |
| None |  |  |  |
| At least one | 0.82 [0.50-1.34] | 0.76 [0.53-1.08] | 1.32 [0.69-2.54] |
| ***Social Networks*** |  |  |  |
| **Network Structure** *^c^*^.^ |  |  |  |
| Family structure | 1.26 [0.36-4.37] | 1.17 [0.59-2.35] | 0.84 [0.30-2.37] |
| Household size | 2.11 [0.68-6.60] | **2.06* [1.06-4.04]** | 1.13 [0.49-2.58] |
| Peer network size | 0.99 [0.88-1.11] | 0.98 [0.92-1.05] | 1.07 [0.99-1.16] |
| Peer network density | 4.68 [0.61-35.9] | 2.51 [0.66-9.60] | 0.15 [0.02-1.58] |
| **Network Function** |  |  |  |
| Family cohesion | **0.38*** [0.25-0.58]** | 0.75 [0.53-1.06] | 0.91 [0.43-1.94] |
| Peer support | 0.67 [0.37-1.24] | 0.94 [0.55-1.63] | **0.37** [0.20-0.68]** |
| School connectedness | 1.03 [0.53-2.01] | 0.68 [0.36-1.30] | 1.46 [0.54-3.99] |
| Neighborhood support | 0.77 [0.14-4.13] | 1.12 [0.33-3.81] | 1.87 [0.43-8.10] |
| **Sex × Network Structure** |  |  |  |
| Female × Married | 1.14 [0.28-4.57] | 0.63 [0.29-1.38] | 0.92 [0.28-3.04] |
| Female × >=4 members | 0.42 [0.11-1.61] | **0.44* [0.19-0.89]** | 1.65 [0.41-6.62] |
| Female × Peer network size | 1.05 [0.91-1.20] | 1.01 [0.93-1.10] | 0.91 [0.81-1.02] |
| Female × Peer network density | 0.08 [0.02-1.71] | 0.46 [0.09-2.70] | 4.35 [0.10-192.90] |
| **Sex × Network Function** |  |  |  |
| Female × Family cohesion | 1.56 [0.91-2.70] | 0.95 [0.64-1.41] | 0.68 [0.27-1.69] |
| Female × Peer support | 1.46 [0.73-2.91] | 0.84 [0.46-1.55] | **2.91* [1.19-7.13]** |
| Female × School connectedness | 0.79 [0.31-2.00] | 1.41 [0.64-3.12] | 0.67 [0.17-2.68] |
| Female × Neighborhood support | 0.89 [0.13-6.35] | 2.10 [0.49-8.97] | 0.85 [0.16-4.45] |
| **Depression** | 4.89*** [3.31-7.24] | 3.03*** [2.27-4.05] | 5.86*** [3.99-8.60] |

*Note.* OR = odds ratio; 95% CI = 95% confidence interval. Cells in bold print indicate significant results for social networks. **p*<0.05; ***p*<0.01; ****p*<0.001

*^a.^* Reference group = Trajectory 1.

*^b^*^.^ Asian, Native American, multiracial and others were collapsed into one group due to small sample sizes.

*^c^*^.^ Family-level network structure included family structure (0 = all other arrangements; 1 = married, two parents) and household size (0 = ≤ 3 people; 1 = ≥ 4 people).

Table A5

*Moderation Effect of Sexual Identity on Social Networks and Suicidal Trajectories^a^*

|  | **Suicidal Ideation** | |
| --- | --- | --- |
|  | **Trajectory 2**  **[*High-decreasing*]** | **Trajectory 3**  **[*Moderate-decreasing-increasing*]** |
|  | OR [95% CI] | OR [95% CI] |
| **Sex** |  |  |
| Male |  |  |
| Female | 1.87** [1.22-2.87] | 0.94 [0.56-1.55] |
| **Sexual Identity** |  |  |
| Heterosexual |  |  |
| Sexual minority | 1.8 [0.032-102.4] | 2.05 [0.19-22.5] |
| **Age [Wave 1]** | 1.02 [0.90-1.16] | 0.97 [0.87-1.07] |
| **Race/Ethnicity***^b.^* |  |  |
| White |  |  |
| Black | 0.34*** [0.19-0.63] | 0.47** [0.27-0.80] |
| Hispanic | 0.50 [0.24-1.06] | 0.66 [0.40-1.07] |
| Others | 0.83 [0.26-2.63] | 0.74 [0.40-1.36] |
| **Maternal Education** |  |  |
| No school/Less than high school |  |  |
| High school or equivalent | 0.91 [0.57-1.47] | 1.09 [0.66-1.78] |
| Some college | 0.73 [0.34-1.58] | 1.49 [0.85-2.62] |
| Graduate school and higher | 1.11 [0.60-2.05] | 0.93 [0.56-1.54] |
| **Public Assistance** |  |  |
| None |  |  |
| At least one | 0.86 [0.53-1.41] | 0.76 [0.53-1.09] |
| ***Social Networks*** |  |  |
| **Network Structure** *^c^*^.^ |  |  |
| Family structure | 1.14 [0.64-2.03] | 0.94 [0.58-1.54] |
| Household size | 1.06 [0.57-1.94] | 1.22 [0.77-1.94] |
| Peer network size | 0.99 [0.93-1.06] | 0.98 [0.93-1.04] |
| Peer network density | 0.75 [0.16-3.46] | 2.15 [0.74-6.24] |
| **Network Function** |  |  |
| Family cohesion | **0.54*** [0.40-0.73]** | **0.70** [0.54-0.91]** |
| Peer support | 0.96 [0.69-1.32] | 0.94 [0.65-1.36] |
| School connectedness | 0.85 [0.56-1.28] | 0.75 [0.50-1.12] |
| Neighborhood support | 1.09 [0.46-2.59] | 1.60 [0.74-3.44] |
| **Sexual Identity × Network Structure** |  |  |
| Sexual minority × Married | 4.12 [0.91-18.6] | 0.87 [0.39-1.92] |
| Sexual minority × >=4 members | 1.10 [0.21-5.68] | 1.13 [0.44-2.90] |
| Sexual minority × Peer network size | 7.74 [0.28-212.2] | 0.36 [0.047-2.78] |
| Sexual minority × Peer network density | **1.19* [1.02-1.40]** | 1.03 [0.93-1.14] |
| **Sexual Identity × Network Function** |  |  |
| Sexual minority × Family cohesion | 1.02 [0.50-2.10] | 1.24 [0.77-2.00] |
| Sexual minority × Peer support | 0.87 [0.37-2.04] | 0.64 [0.37-1.12] |
| Sexual minority × School connectedness | 0.76 [0.24-2.40] | 1.49 [0.74-2.99] |
| Sexual minority × Neighborhood support | **0.10** [0.02-0.57]** | 1.21 [0.31-4.66] |
| **Depression** | 5.06*** [3.45-7.40] | 3.01*** [2.27-3.99] |

*Note.* OR = odds ratio; 95% CI = 95% confidence interval. Cells in bold print indicate significant results for social networks. **p*<0.05; ***p*<0.01; ****p*<0.001

*^a.^* Reference group = Trajectory 1.

*^b^*^.^ Asian, Native American, multiracial and others were collapsed into one group due to small sample sizes.

*^c^*^.^ Family-level network structure included family structure (0 = all other arrangements; 1 = married, two parents) and household size (0 = ≤ 3 people; 1 = ≥ 4 people).
