# Supplementary material for: Adolescent social networks matter for suicidal trajectories: disparities across race/ethnicity, sex, sexual identity, and socioeconomic status
Source: Psychol Med. 2021 Mar 3;52(15):3677–88. doi: 10.1017/S0033291721000465 (PMC9772914; doi:10.1017/S0033291721000465)
Supplement: Supplementary file 1 [file S0033291721000465sup.zip › S0033291721000465sup003.docx]

# Figure A1

###
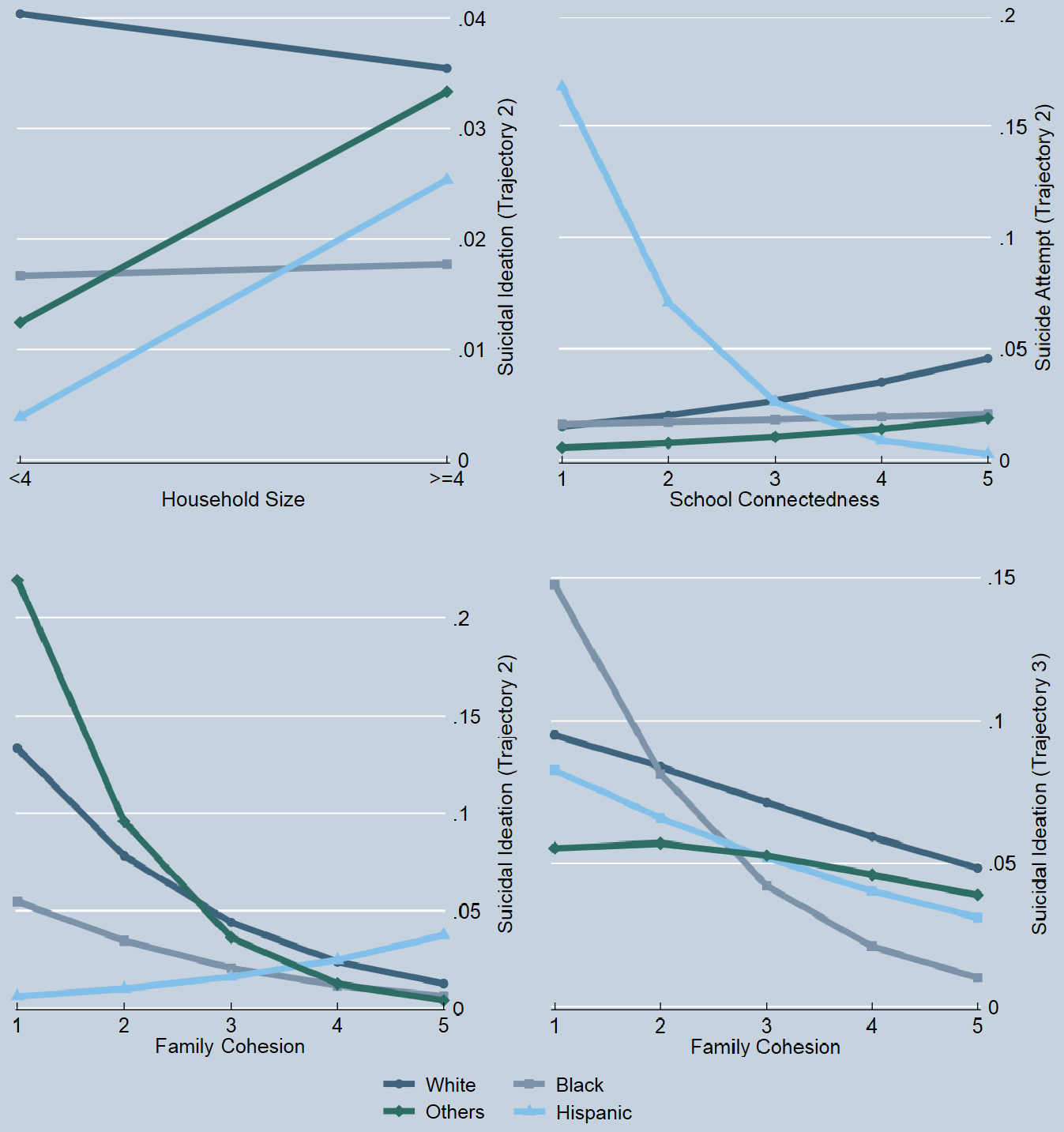
Moderation Effect of Race/ethnicity on the Association between Social Networks and Suicidal Trajectories
