# Supplementary figures and images for: Adolescent social networks matter for suicidal trajectories: disparities across race/ethnicity, sex, sexual identity, and socioeconomic status
Source: Psychol Med. 2021 Mar 3;52(15):3677–88. doi: 10.1017/S0033291721000465 (PMC9772914; doi:10.1017/S0033291721000465)

Figure A2

*Moderation Effect of Sex and Sexual Identity on the Association*


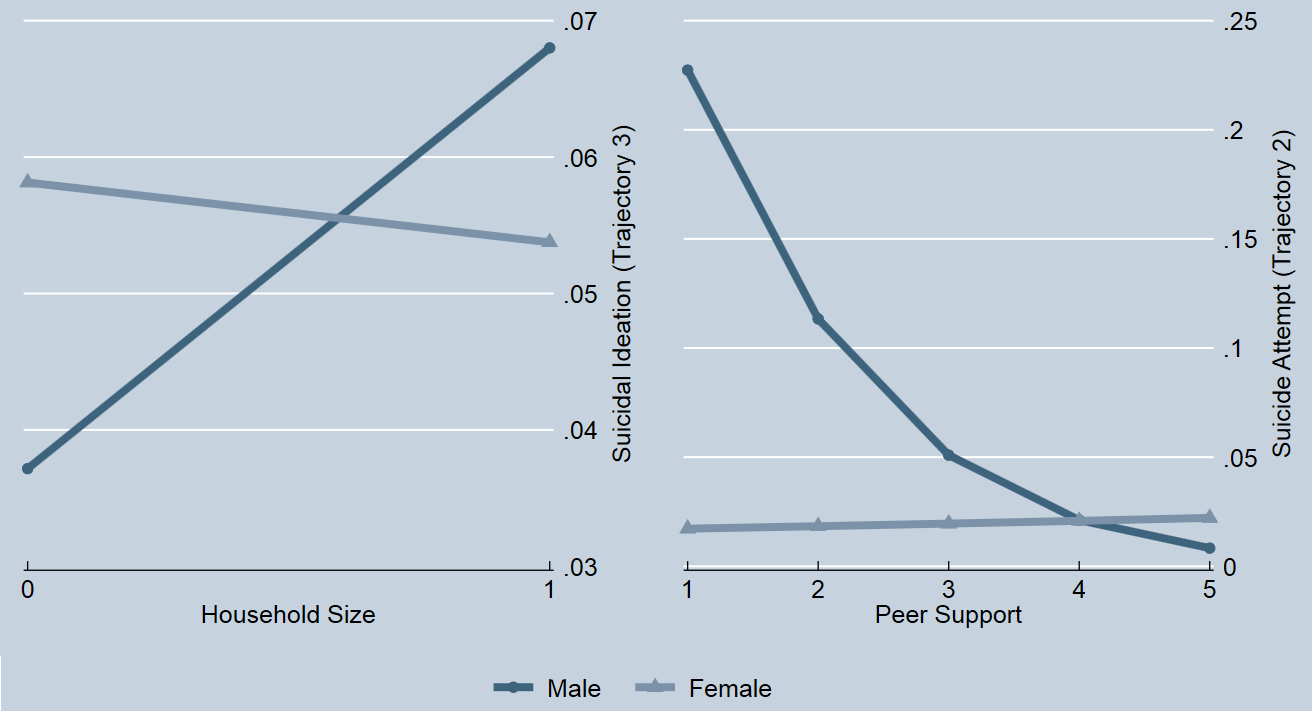

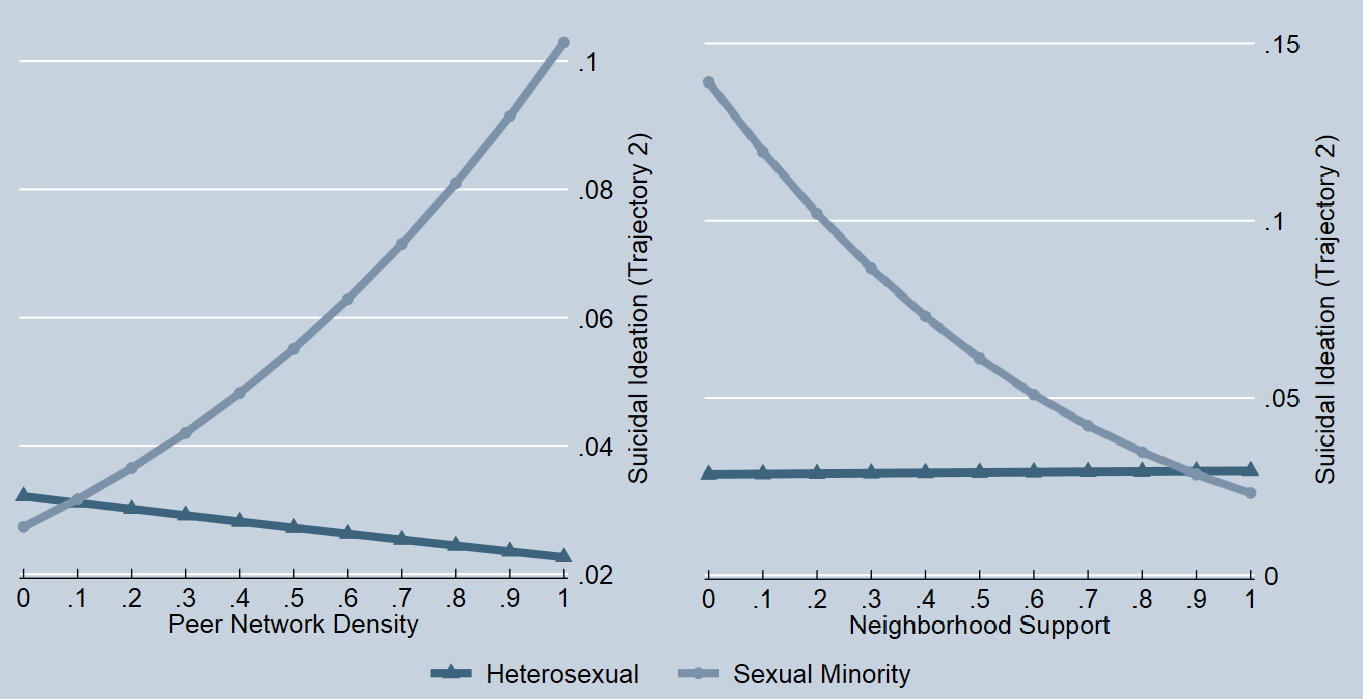

Supplement: Supplementary file 1 [file S0033291721000465sup.zip › S0033291721000465sup002.docx]
